# Adverse events in both childhood and adulthood are associated with molecular, clinical and functional markers of ageing

**DOI:** 10.1186/s12916-026-04815-x

**Published:** 2026-04-28

**Authors:** Monica Aas, Thole H Hoppen, Nexhmedin Morina, Shiyu Zhang, Bin Li, Vid Mlakar, Julian Mutz

**Affiliations:** 1 Social, Genetic and Developmental Psychiatry Centre, Institute of Psychiatry, Psychology & Neuroscience, King’s College London, London, UK; 2 Institute of Psychology, University of Münster, Münster, Germany; 3 National Clinical Research Center for Geriatric Disorders, Department of Geriatrics, Xiangya Hospital & Center for Medical Genetics, School of Life Sciences, Central South University, Changsha, China

**Keywords:** Childhood adversity, Adulthood adversity, Trauma, Biological ageing, Metabolomics, Frailty, Telomere length, Grip strength, UK Biobank, Life-course exposure

## Abstract

**Background:**

Adverse events across the lifespan have been linked to poorer health outcomes, but the biological mechanisms remain unclear. The aim of this study was to quantify the independent and joint associations of adversity experienced in childhood and/or adulthood with molecular, clinical and functional markers of biological ageing.

**Methods:**

We analysed data from up to 153,557 middle-aged and older adults in the UK Biobank. Adversity was assessed through questionnaires capturing five types of childhood and adulthood adverse events. Biological ageing markers included metabolomic age (MileAge) delta, a metabolomic mortality profile, the frailty index, telomere length and grip strength. Regression models were adjusted for age, sex, education, income, ethnicity and neighbourhood deprivation.

**Results:**

Across childhood and adulthood exposures, adversity and its severity were most consistently associated with higher frailty index values. The strongest associations were observed in individuals exposed to multiple types of adverse events. Individuals who experienced adversity in both childhood and adulthood also had a metabolite-predicted age exceeding their chronological age and lower grip strength. Abuse was more consistently associated with biological ageing markers than neglect.

**Conclusions:**

Cumulative exposure to adversity across childhood and adulthood is associated with older biological ageing profiles across multiple domains. These findings highlight biological ageing as a potential pathway linking adversity to poor health outcomes and premature mortality.

**Supplementary Information:**

The online version contains supplementary material available at 10.1186/s12916-026-04815-x.

## Background

Adverse events occurring in both childhood and adulthood have been linked to detrimental health outcomes in later life [1–4]. Chronic or repeated exposure to adverse events is linked to accelerated biological ageing [5, 6]. However, the mechanisms underlying this relationship remain poorly understood. Recent findings suggest that biological ageing can be accelerated by severe stress exposure and may be restored upon recovery [7], suggesting that both timing and length of exposure are crucial.

The availability of high-throughput molecular data has enabled the development of diverse markers of biological ageing. A promising approach for developing such markers is metabolomics, which captures the interplay between environmental exposures and genetics. The metabolome bridges the gap between genotype and phenotype [8], providing insights into cellular processes linked to health and disease. Indeed, metabolomics can identify actionable targets for intervention [9]. However, studies investigating the relationship between exposure to adverse events and metabolomic markers of ageing remain scarce.

Frailty is a clinical syndrome characterised by reduced physiological reserve and increased vulnerability to adverse health outcomes, including mortality [10]. It is often measured using a frailty index, which quantifies the proportion of accumulated health deficits (e.g., symptoms, signs or diseases) that an individual has. Due to demographic changes towards population ageing, frailty is becoming an emerging global health burden. However, it also represents a potentially modifiable target for intervention to extend healthy life expectancy. A recent study of 862 older adults found greater frailty in those with higher stress levels [11], indicating that stress exposure may contribute to frailty.

Telomeres are structures at the ends of chromosomes made of repeating TTAGGG nucleotides [12]. Their role is to guard DNA from cellular damage. When telomeres become critically short, the risk of apoptosis is increased and proliferation is arrested, thus compromising tissue renewal capacity and function [13]. Telomere length is an indicator of cellular replicative history and shorter telomeres are associated with an older chronological age [12]. Increasing evidence links childhood adversity to accelerated telomere shortening [14, 15]. However, most studies have been conducted in small samples or clinical populations, limiting generalisability.

Grip strength is a well-established functional marker of ageing, predictive of morbidity and mortality [16]. A recent study based on 879 older adults reported that stressful life events in childhood were associated with lower muscle strength [17]. The authors proposed that impaired skeletal muscle mitochondrial energetics might represent a potential biological pathway through which early-life adversity may impact health later in life.

Population-scale analyses in the UK Biobank have begun to characterise associations between childhood adversity and markers of biological ageing and age-related health outcomes. At the systemic level, childhood adversity has been linked to higher allostatic load in females [18] and to clinical biomarker-based ageing measures, such as PhenoAge and Klemera-Doubal biological age, exceeding chronological age [19, 20]. Childhood adversity is also associated with greater multimorbidity [21] and higher odds of frailty [21, 22]. Studies of molecular markers suggest that childhood adversity is associated with shorter telomeres, particularly amongst individuals exposed to two or more adversity types, with physical and sexual abuse also being individually associated [23]. Associations with telomere length were identified primarily in females, with only physical abuse associated in males [24]. Childhood adversity has been linked to older facial age and shorter telomeres relative to genetically predicted length only when co-occurring with psychopathology [25]. Finally, individuals exposed to multiple types of childhood adversity also exhibit weaker grip strength [26].

Despite this rapidly expanding literature, most UK Biobank studies have focused on childhood adversity and far fewer have investigated adversity in adulthood or cumulatively across childhood and adulthood. Two recent analyses indicate that both childhood and adulthood adversity, especially when co-occurring, are associated with higher cardiovascular disease risk [27], and that biological ageing may partially mediate these associations [28]. To our knowledge, no prior studies have examined associations between adversity, whether in childhood, adulthood or both, and NMR-based metabolomic ageing markers.

Here, we use data from the UK Biobank to investigate associations between exposure to adverse events in childhood and adulthood and multiple, complementary markers of biological ageing. We selected outcomes spanning distinct but correlated aspects of ageing: two metabolomic composites (a metabolomic clock trained on chronological age, MileAge delta, and a mortality-trained metabolomic risk profile), a clinical measure of age-related health deficit accumulation (frailty index), a cellular marker (telomere length) and a functional measure (grip strength). Assessing these outcomes jointly allows triangulation across molecular, clinical and functional levels, helping to identify which ageing processes adversity most strongly relates to. Extending prior studies [20–23], we evaluate these markers in a large, well-characterised sample to provide a cross-domain test of the link between adversity and midlife ageing. We further provide new insights into the timing (i.e., childhood, adulthood or both), breadth and severity (i.e., overall number and intensity of adversity) and types (e.g., emotional abuse or physical neglect) of adverse events. We hypothesised that individuals exposed to adversity in both childhood and adulthood would have older biological ageing profiles compared with those exposed to adversity only in childhood, only in adulthood, or not at any point. We did not aim to test specific mechanistic pathways linking adversity to biological ageing but to assess whether midlife differences in ageing markers are observable by adversity exposure. Multiple biological and behavioural processes, including inflammation, stress physiology and metabolic dysregulation, may contribute to these links.

## Methods

### Study population

The UK Biobank recruited over 500,000 individuals from England, Scotland and Wales between 2006 and 2010. At baseline, participants completed health and sociodemographic questionnaires, underwent physical examinations and provided biological samples. All variables used in this study and their UK Biobank field IDs are listed in Additional file 1: Table S1 to facilitate transparency.

### Adversity and trauma

Exposure to adversity and trauma was retrospectively assessed via the UK Biobank online mental health questionnaire (MHQ) between 2016 and 2017 [29]. The median interval between the baseline assessment and MHQ completion was 7.57 years (IQR = 1.38).

Adverse events in childhood were assessed using the Childhood Trauma Screener [30], a short version of the Childhood Trauma Questionnaire [31]. It comprises five items, with response options “never true”, “rarely true”, “sometimes true”, “often” and “very often true” (5-point Likert): “When I was growing up, … 1) I felt loved [emotional neglect]; 2) people in my family hit me so hard that it left me with bruises or marks [physical abuse]; 3) I felt that someone in my family hated me [emotional abuse]; 4) someone molested me sexually [sexual abuse]; and 5) there was someone to take me to the doctor if I needed it [physical neglect].” Individuals were classified as having experienced adversity or trauma in childhood (yes/no) based on the following cut-offs: physical abuse, emotional abuse or sexual abuse (“rarely true”, “sometimes true”, “often” or “very often true”); emotional neglect or physical neglect (“rarely true” or “sometimes true”; item reverse coded). We also derived a sum score (ranging from 0 to 5) from these binary indicators, reflecting the overall childhood trauma burden (breadth of exposure). To capture severity, we further derived a weighted sum score ranging from 0 to 15. For this, responses were recorded on a scale from 0 to 3 reflecting the severity of the item (0 = no trauma, 1 = mild trauma, 2 = moderate trauma, 3 = severe trauma), based on severity and frequency of the trauma. The coding scheme for the weighted sum score is published in Pitharouli et al. [32].

Adverse events in adulthood were assessed using questions adapted from the 2010/2011 British Crime Survey questions to identify victims of crime and domestic violence [33]. Five questions were asked, with response options “never true”, “rarely true”, “sometimes true”, “often” and “very often true”: “Since I was sixteen, … 1) I have been in a confiding relationship [emotional neglect]; 2) a partner or ex-partner repeatedly belittled me to the extent that I felt worthless [emotional abuse]; 3) a partner or ex-partner deliberately hit me or used violence in any other way [physical abuse]; 4) a partner or ex-partner sexually interfered with me, or forced me to have sex against my wishes [sexual abuse]; 5) there was money to pay the rent or mortgage when I needed it [economic hardship].” Individuals were classified as having experienced adversity or trauma in adulthood (yes/no) based on the following cut-offs: emotional abuse, sexual abuse or physical violence (“rarely true”, “sometimes true”, “often” or “very often true”); emotional neglect (“rarely true” or “sometimes true”; reverse coded); economic hardship (“rarely true”, “sometimes true” or “often”; reverse coded) [29]. We also derived a sum score (ranging from 0 to 5) from these binary indicators, reflecting overall adulthood trauma burden (breadth of exposure).

### Metabolomic age (MileAge) delta

Nuclear magnetic resonance (NMR) spectroscopy–derived metabolomic biomarkers were quantified in non-fasting plasma samples using the Nightingale Health platform, which ascertains 249 biomarkers (168 in absolute concentrations and 81 derived ratios) [34]. Removal of technical variation was performed using the “ukbnmr” R package (algorithm v2) [35]. In a prior study [36], we developed a metabolomic ageing clock using a Cubist rule-based regression model. Individual-level age predictions were obtained by aggregating the predictions from the ten test sets of the outer loop of the nested cross-validation to avoid potential overfitting. Metabolomic age (MileAge) delta represents the age-bias adjusted difference between metabolite-predicted and chronological age, with positive values indicating older biological ageing profiles [36].

### Metabolomic mortality profile score

In a previous study [37], we developed a metabolomic mortality profile score. Complementing the 249 biomarkers provided by the Nightingale Health platform, 76 additional lipid, cholesterol and fatty acid ratios were derived [35], resulting in a total of 325 biomarkers. A Least Absolute Shrinkage and Selection Operator (LASSO) Cox proportional hazards model predicting all-cause mortality was developed in English and Welsh participants (*N* = 234,553). We derived a metabolomic mortality profile score in an out-of-sample dataset of Scottish participants (*N* = 15,788) as the linear combination of the 54 biomarkers with non-zero coefficients in the LASSO Cox model weighted by their log hazard ratio for all-cause mortality. We used the subset of participants from Scotland for validation to avoid overfitting and because demographic and health-related characteristics vary across the different regions of the UK [38]. Higher scores indicate an elevated mortality risk.

### Frailty index

A frailty index was derived from health deficits reported via touch-screen questionnaires or during nurse-led interviews that met the following criteria: indicators of poor health, more prevalent in older individuals, neither rare nor universal, covering multiple areas of functioning and available for ≥ 80% of participants [39]. The 49 items that met those criteria included cardiometabolic, cranial, immunological, musculoskeletal, respiratory and sensory traits, well-being, infirmity, cancer and pain. Categorical variables were dichotomised (deficit absent = zero; deficit present = one) and ordinal variables were mapped onto a score between zero and one. The sum of deficits was divided by the number of possible deficits, resulting in frailty index scores between zero and one, with higher scores indicating greater levels of frailty [10]. Participants with missing data for ≥ 10/49 variables were excluded [39].

### Telomere length

Leukocyte telomere length was measured using a quantitative polymerase chain reaction (qPCR) assay that expresses telomere length as the ratio of the telomere repeat copy number (T) relative to a single-copy gene (S) encoding haemoglobin subunit beta [40]. The T/S ratio is proportional to average telomere length [41]. Measurements were adjusted for operational and technical parameters (PCR machine, staff member, enzyme batch, primer batch, temperature, humidity, primer batch × PCR machine, primer batch × staff member, A260/A280 ratio of the DNA sample and A260/A280 ratio squared), log_*e*_ transformed and *Z*-standardised.

### Grip strength

Maximal grip strength in whole kilogram-force units was measured using a Jamar J00105 hydraulic hand dynamometer (measurement range 0–90 kg) for both hands. We used the maximal grip strength of the participant’s self-reported dominant hand. If no data on handedness were available, we used the highest value.

### Covariates

Potential confounders included chronological age, sex, highest educational or professional qualification, gross annual household income, ethnicity and neighbourhood deprivation (assessed via the Townsend deprivation index).

### Exclusion criteria

Individuals with missing data or who responded “prefer not to answer” to any questionnaire items on adverse events were excluded from the derived variables (binary phenotypes and sum scores). For item-specific analyses, only those with missing data or with a “prefer not to answer” response for the specific item were excluded. For categorical covariates, “do not know” responses, “prefer not to answer” responses and missing data were coded as a missing level. For the analyses of MileAge delta, we applied exclusions as per the original study [36]: women with possible pregnancy, as metabolite profiles differ during pregnancy; individuals with discordant genetic and self-reported sex; and individuals with missing or outlier metabolite values (4 × the interquartile range from the median).

### Statistical analyses

Data processing, analyses and visualisations were performed in R (version 4.3.0). Sample characteristics were summarised using means and standard deviations or counts and percentages. Associations between adverse events (exposures) and MileAge delta, the metabolomic mortality profile score, the frailty index, telomere length and grip strength (outcomes) were estimated using ordinary least squares regression. All outcomes were scaled to have a mean equal to zero and a standard deviation of one, allowing for direct comparison of the association estimates. Telomere length and grip strength were reverse coded prior to analysis. For the analysis of adverse childhood events, we examined a binary (yes/no) exposure definition, unweighted and weighted sum scores as well as an ordinal definition of exposure (based on the unweighted sum score). For adverse events in adulthood, we examined a binary (yes/no) exposure definition, an unweighted sum score and an ordinal definition (based on the sum score). We further examined, for both child and adulthood adversity, whether exposure to multiple traumatic events (two or more) was associated with biological ageing markers. Independent and joint associations were quantified using a cross-classification of childhood and adulthood adverse events (childhood only, adulthood only, both or neither), derived from the binary exposure definitions described above. This approach captures shared and unique associations of adversity across life stages. To investigate whether specific types of adverse events were associated with markers of biological ageing, we further examined associations for each individual item included in the questionnaires. For each outcome, we fitted a minimally adjusted model including chronological age and sex (Model 1) and a fully adjusted model also including education, income, ethnicity and deprivation (Model 2). Given the large sample size, we report standardised beta coefficients with 95% confidence intervals alongside *p*-values. *P*-values were adjusted for multiple testing using the Benjamini–Hochberg correction method, with a two-tailed test and a false discovery rate of 5%. Given the exploratory nature of the study, we did not predefine formal primary and secondary hypotheses. To balance the risks of false positives and over-adjustment, corrections were applied within pre-specified families of related tests (see table legends for details). We did not adopt a single global correction across all tests because many analyses involve correlated tests of different parameterisations of the same exposure, and a global adjustment would therefore be inappropriate and overly conservative, inflating the risk of false negatives.

## Results

### Adverse events in childhood

**Fig. 1:**
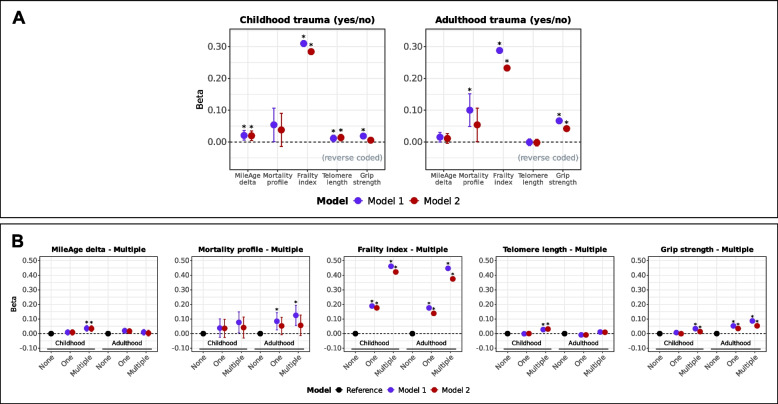
**A** Ageing markers and trauma (yes/no). Associations between trauma (adverse/traumatic events in childhood and in adulthood) and ageing markers (MileAge delta, metabolomic mortality profile, frailty index, telomere length [reverse coded] and grip strength [reverse coded]). Asterisks indicate statistically significant associations, after correcting p-values for multiple testing using the Benjamini–Hochberg procedure (across ageing markers and models, separately for childhood and adulthood exposures). **B** Ageing markers and multiple traumas. Associations between multiple traumas (adverse/traumatic events in childhood and in adulthood) and ageing markers (MileAge delta, metabolomic mortality profile, frailty index, telomere length [reverse coded] and grip strength [reverse coded]). Asterisks indicate statistically significant associations, after correcting p-values for multiple testing using the Benjamini–Hochberg procedure (across exposure levels and models, separately for each ageing marker and childhood and adulthood exposures). **A**-**B** Estimates shown are ordinary least squares regression beta coefficients and 95% confidence intervals. Model 1–adjusted for chronological age and sex; Model 2–adjusted for chronological age, sex, ethnicity, highest educational/professional qualification, annual gross household income and Townsend deprivation index. Sample sizes reported in Tables S2 and S7

Amongst 153,557 participants, 63,066 (41%) reported experiencing at least one adverse event during childhood (Additional file 1: Table S2). Analytical sample sizes are shown in Additional file 1: Table S3; those include individuals with/without adulthood adversity exposure. Childhood adverse events (yes/no) were associated with a metabolite-predicted age exceeding chronological age (*β* = 0.020, 95% CI 0.005–0.035, *p* = 0.018), higher frailty index values (*β* = 0.284, 95% CI 0.276–0.293, *p* < 0.001) and shorter telomeres (*β* = 0.014, 95% CI 0.004–0.024, *p* = 0.015) (Fig. 1A).

### Associations with adversity burden in childhood

**Fig. 2:**
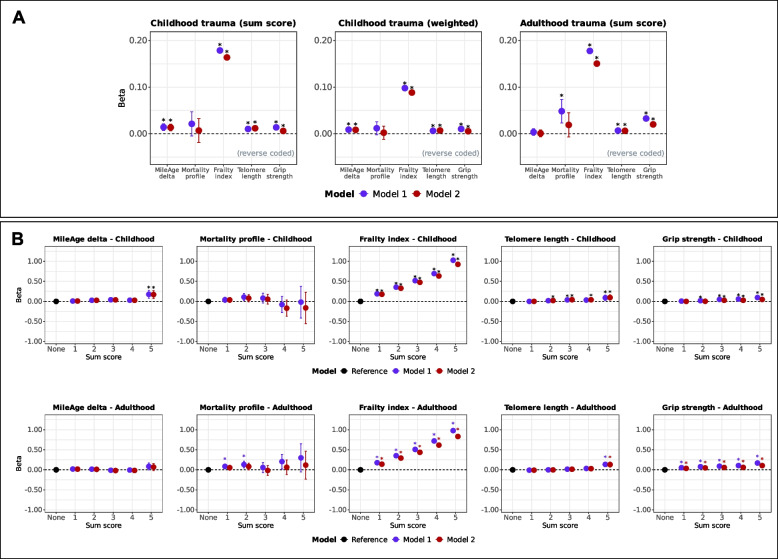
**A** Ageing markers and trauma (sum scores). Associations between trauma (adverse/traumatic events in childhood [unweighted and weighted sum scores] and in adulthood) and ageing markers (MileAge delta, metabolomic mortality profile, frailty index, telomere length [reverse coded] and grip strength [reverse coded]). Asterisks indicate statistically significant associations, after correcting p-values for multiple testing using the Benjamini–Hochberg procedure (across ageing markers and models, separately for each sum score). **B** Ageing markers and trauma (sum scores, categorical). Associations between trauma (adverse/traumatic events in childhood and in adulthood) and ageing markers (MileAge delta, metabolomic mortality profile, frailty index, telomere length [reverse coded] and grip strength [reverse coded]). Asterisks indicate statistically significant associations, after correcting p-values for multiple testing using the Benjamini–Hochberg procedure (across exposure levels and models, separately for each ageing marker and childhood and adulthood exposures). **A**-**B** Estimates shown are ordinary least squares regression beta coefficients and 95% confidence intervals. Model 1–adjusted for chronological age and sex; Model 2–adjusted for chronological age, sex, ethnicity, highest educational/professional qualification, annual gross household income and Townsend deprivation index. Sample sizes reported in Tables S2 and S7

Exposure to multiple (two or more) adverse events in childhood was associated with differences in all biological ageing markers except the metabolomic mortality profile (Fig. 1B; Tables S4). Analyses using both unweighted (breadth) and weighted (breadth and severity) sum scores indicated that greater adversity burden was associated with older biological ageing profiles across markers, except the metabolomic mortality profile (Table 2). The strongest associations were observed for the frailty index (unweighted: *β* = 0.164, 95% CI 0.160–0.168, *p* < 0.001; weighted: *β* = 0.088, 95% CI 0.086–0.091, *p* < 0.001) (Fig. 2A). Although two, three or five adverse events were nominally associated with a MileAge exceeding chronological age (Additional file 1: Table S5), only sum scores of five remained statistically significant after multiple testing corrections (*β* = 0.170, 95% CI 0.073–0.267, *p* = 0.003). There was no evidence that any number of adverse events was associated with the metabolomic mortality profile. We observed a graded, approximately linear association between the number of adverse events and higher frailty index values (*β* = 0.177, 95% CI 0.166–0.187, *p* < 0.001 to *β* = 0.925, 95% CI 0.869–0.981, *p* < 0.001) (Fig. 2B). Exposure to two or more or three or more adverse events was associated with shorter telomeres and lower grip strength after full adjustment.

### Specific types of adverse events in childhood

Childhood emotional abuse, but not neglect, was associated with higher MileAge delta (*β* = 0.036, 95% CI 0.015–0.056, *p* = 0.002) (Additional file 1: Table S6). No statistically significant associations with the metabolomic mortality profile were observed after multiple testing corrections. All types of childhood adversity were associated with higher frailty index scores, and, except for physical neglect, with shorter telomeres (Fig. 4). Most childhood adverse events were associated with lower grip strength; however, sexual abuse was not statistically significant after full adjustment, and physical abuse was associated with higher grip strength (*β* = − 0.022, 95% CI − 0.030 to − 0.014, *p* < 0.001).

### Adverse events in adulthood

Amongst 150,848 participants, 80,895 (53%) reported at least one adverse event during adulthood (Additional file 1: Table S7). Analytical sample sizes are reported in Additional file 1: Table S8; those include individuals with/without childhood adversity exposure. Adulthood adversity (yes/no) was associated with higher frailty index values (*β* = 0.233, 95% CI 0.224–0.242, *p* < 0.001) and lower grip strength (*β* = 0.042, 95% CI 0.035–0.049, *p* < 0.001) (Fig. 1A). The association between adulthood adverse events and higher metabolomic mortality profile scores did not survive multiple testing corrections after full adjustment (*p* = 0.065). No associations were found with MileAge delta or telomere length (Table 1).

**Table 1:** Associations between adverse/traumatic events and ageing markers (binary)

	Model 1 (adj. age and sex)				Model 2 (full adjustment)			
	*β*	95% CI		*p*	*β*	95% CI		*p*
**Childhood**								
MileAge delta	0.021	0.006	0.036	0.015	0.020	0.005	0.035	0.018
Metabolomic profile	0.054	0.001	0.107	0.055	0.038	− 0.014	0.090	0.152
Frailty index	0.310	0.301	0.319	< 0.001	0.284	0.276	0.293	< 0.001
Telomere length	0.012	0.001	0.022	0.037	0.014	0.004	0.024	0.015
Grip strength	0.019	0.012	0.025	< 0.001	0.006	− 0.001	0.012	0.104
**Adulthood**								
MileAge delta	0.015	0.000	0.030	0.065	0.011	− 0.004	0.027	0.191
Metabolomic profile	0.100	0.048	0.152	< 0.001	0.054	0.002	0.107	0.065
Frailty index	0.288	0.279	0.297	< 0.001	0.233	0.224	0.242	< 0.001
Telomere length	− 0.001	− 0.011	0.010	0.901	− 0.002	− 0.012	0.008	0.793
Grip strength	0.067	0.060	0.073	< 0.001	0.042	0.035	0.049	< 0.001

CI = confidence interval. Model 1–adjusted for chronological age and sex; Model 2–adjusted for chronological age, sex, ethnicity, highest educational/professional qualification, annual gross household income and Townsend deprivation index. *P*-values shown are corrected for multiple testing using the Benjamini–Hochberg procedure (across ageing markers and models, separately for childhood and adulthood). Sample sizes reported in Tables S2 and S7

### Associations with adversity burden in adulthood

Exposure to multiple adverse events in adulthood was associated with higher metabolomic mortality profile scores, higher frailty index values and lower grip strength (Fig. 1B). However, the associations with the metabolomic mortality profile were not statistically significant after full adjustment (Tables S9). Analyses of unweighted sum scores (reflecting breadth of adversity exposure) indicated that greater adversity burden in adulthood was associated with all biological ageing markers except MileAge delta (*p* = 0.796) and with the metabolomic mortality profile only in the age and sex-adjusted model (Table 2). The strongest association was observed for the frailty index (*β* = 0.150, 95% 0.146–0.155, *p* < 0.001) (Fig. 2A). There was no evidence that any number of adverse events was associated with MileAge delta after multiple testing correction (Additional file 1: Table S10). Sum scores of one or two were associated with higher metabolomic mortality profile scores after multiple testing correction in the age and sex-adjusted model (*β* = 0.085, 95% CI 0.025–0.144, *p* = 0.027 and *β* = 0.131, 95% CI 0.048–0.213, *p* = 0.020). A graded, approximately linear association was observed between the number of adverse events and higher frailty index values (*β* = 0.140, 95% CI 0.130–0.150, *p* < 0.001 to *β* = 0.831, 95% CI 0.780–0.882, *p* < 0.001) (Fig. 2B). A sum score of five was associated with shorter telomeres (*β* = 0.130, 95% CI 0.072–0.189, *p* < 0.001). A greater number of adverse events was consistently associated with lower grip strength (*β* = 0.105, 95% CI 0.067–0.143, *p* < 0.001 for a sum score of five).

**Table 2:** Associations between adverse/traumatic events and ageing markers (sum score)

	Model 1 (adj. age and sex)				Model 2 (full adjustment)			
	*β*	95% CI		*p*	*β*	95% CI		*p*
**Childhood**								
MileAge delta	0.014	0.007	0.021	< 0.001	0.014	0.006	0.021	< 0.001
Metabolomic profile	0.021	− 0.005	0.047	0.119	0.007	− 0.019	0.033	0.598
Frailty index	0.179	0.174	0.183	< 0.001	0.164	0.160	0.168	< 0.001
Telomere length	0.010	0.006	0.015	< 0.001	0.012	0.007	0.017	< 0.001
Grip strength	0.014	0.011	0.017	< 0.001	0.006	0.003	0.009	< 0.001
**Childhood (weighted)**								
MileAge delta	0.009	0.005	0.013	< 0.001	0.008	0.005	0.012	< 0.001
Metabolomic profile	0.012	− 0.002	0.026	0.110	0.002	− 0.012	0.016	0.753
Frailty index	0.098	0.096	0.100	< 0.001	0.088	0.086	0.091	< 0.001
Telomere length	0.006	0.004	0.009	< 0.001	0.007	0.005	0.010	< 0.001
Grip strength	0.010	0.009	0.012	< 0.001	0.005	0.004	0.007	< 0.001
**Adulthood**								
MileAge delta	0.004	− 0.003	0.011	0.352	0.001	− 0.006	0.008	0.796
Metabolomic profile	0.048	0.023	0.074	< 0.001	0.019	− 0.007	0.045	0.187
Frailty index	0.178	0.173	0.182	< 0.001	0.150	0.146	0.155	< 0.001
Telomere length	0.007	0.002	0.012	0.008	0.006	0.001	0.011	0.016
Grip strength	0.033	0.030	0.036	< 0.001	0.020	0.017	0.023	< 0.001

CI = confidence interval. Model 1–adjusted for chronological age and sex; Model 2–adjusted for chronological age, sex, ethnicity, highest educational/professional qualification, annual gross household income and Townsend deprivation index. *P*-values shown are corrected for multiple testing using the Benjamini–Hochberg procedure (across ageing markers and models, separately for each sum score). Sample sizes reported in Tables S2 and S7

### Specific types of adverse events in adulthood

**Fig. 3:**
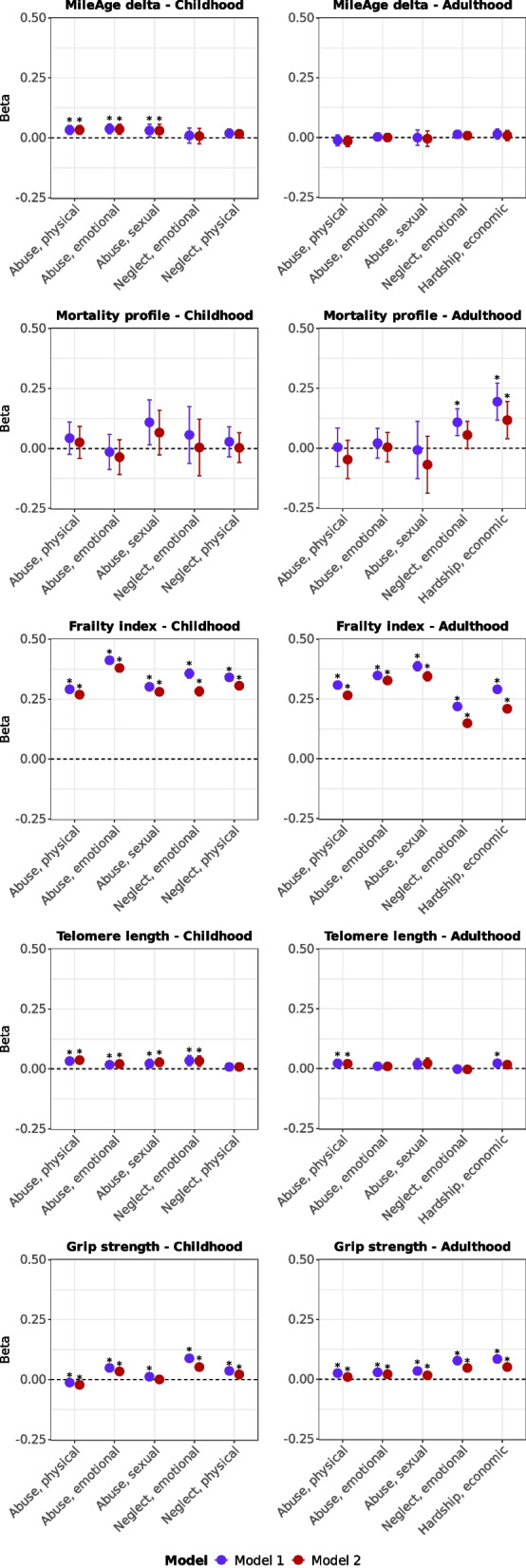
Ageing markers and trauma (item-specific). Associations between specific trauma items (adverse/traumatic events in childhood and in adulthood) and ageing markers (MileAge delta, metabolomic mortality profile, frailty index, telomere length [reverse coded] and grip strength [reverse coded]). Estimates shown are ordinary least squares regression beta coefficients and 95% confidence intervals. Model 1–adjusted for chronological age and sex; Model 2–adjusted for chronological age, sex, ethnicity, highest educational/professional qualification, annual gross household income and Townsend deprivation index. Asterisks indicate statistically significant associations, after correcting p-values for multiple testing using the Benjamini–Hochberg procedure (across trauma items and models, separately for each ageing marker and childhood and adulthood exposures). Childhood and adulthood items with similar labels are distinct measures and not repeated measures of the same construct across time. Sample sizes reported in Tables S2 and S7

There was no evidence that any type of adulthood adverse event was associated with MileAge delta (Additional file 1: Table S11). Emotional neglect and economic hardship were associated with higher metabolomic mortality profile scores, but only economic hardship remained statistically significant after full adjustment (*β* = 0.117, 95% CI 0.039–0.195, *p* = 0.011). All adulthood adverse events were associated with higher frailty index values (Fig. 3). These associations were stronger for abuse than for emotional neglect or economic hardship (e.g., *β* = 0.149, 95% CI 0.139–0.158, *p* < 0.001 and *β* = 0.327, 95% CI 0.317–0.338, *p* < 0.001 for emotional neglect compared to emotional abuse, respectively). Only physical abuse remained associated with shorter telomeres after full adjustment and multiple testing corrections (*β* = 0.020, 95% CI 0.005–0.035, *p* = 0.025). All adulthood adversity types were associated with lower grip strength.

### Cross-classification of adverse events in childhood and adulthood

**Fig. 4:**
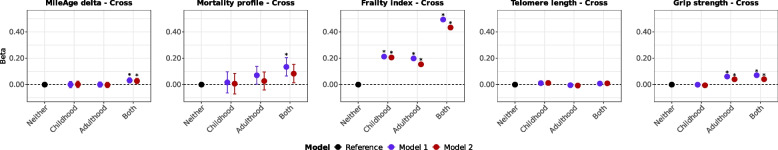
Ageing markers and trauma (cross-classification child and adulthood). Associations between trauma (adverse/traumatic events in childhood and/or adulthood) and ageing markers (MileAge delta, metabolomic mortality profile, frailty index, telomere length [reverse coded] and grip strength [reverse coded]). Estimates shown are ordinary least squares regression beta coefficients and 95% confidence intervals. Model 1–adjusted for chronological age and sex; Model 2–adjusted for chronological age, sex, ethnicity, highest educational/professional qualification, annual gross household income and Townsend deprivation index. Asterisks indicate statistically significant associations, after correcting p-values for multiple testing using the Benjamini–Hochberg procedure (across exposure levels and models, separately for each ageing marker). Sample sizes reported in Table S11

A subset of 40,086 (27.1%) individuals experienced adverse events in both childhood and adulthood (Additional file 1: Table S12). Exposure to adversity in childhood only, adulthood only or in both childhood and adulthood was associated with higher frailty index values (Fig. 4). Associations were stronger in childhood than adulthood adversity (e.g., *β* = 0.206, 95% CI 0.192–0.220, *p* < 0.001 versus *β* = 0.154, 95% CI 0.143–0.166, *p* < 0.001 for the frailty index, respectively) and greatest amongst individuals exposed to both (*β* = 0.434, 95% CI 0.422–0.445, *p* < 0.001). Only individuals exposed to adversity in both childhood and adulthood had a metabolite-predicted age exceeding chronological age (*β* = 0.028, 95% CI 0.008–0.048, *p* = 0.021) (Additional file 1: Table S13). No associations with the metabolomic mortality profile were statistically significant after full adjustment and multiple testing corrections. Exposure to adverse events in adulthood only or in both childhood and adulthood was associated with lower grip strength, of comparable magnitude. No other associations between adversity and ageing markers (telomere length, MileAge delta, metabolomic mortality profile or grip strength) survived corrections for multiple testing. An overview of all results is shown in Additional file 1: Table S14.

## Discussion

In this large, well-characterised sample of middle-aged and older adults, we investigated whether adverse events experienced in childhood and/or adulthood were associated with molecular, clinical and functional markers of biological ageing. This study was not designed to identify underlying mechanisms but to test whether adversity is associated with measurable differences in midlife biological ageing across domains. We found that individuals who experienced both childhood and adulthood adversity had a metabolite-predicted age (MileAge) exceeding their chronological age, greater frailty and lower grip strength compared to those who experienced adversity in only one life stage or not at any point. Associations with shorter telomeres were observed for childhood adversity, particularly experiences of abuse. By analysing molecular, clinical and functional ageing markers together, we provide an integrated view of how adversity maps onto different levels of the biological ageing process rather than focusing on a single biomarker.

Our findings are broadly consistent with prior UK Biobank studies linking childhood adversity to advanced biological ageing. For example, earlier work has shown that adversity is associated with higher allostatic load [18], clinical biomarker-based ageing indices exceeding chronological age [19, 20], greater multimorbidity and frailty [21, 22], shorter telomeres in those exposed to multiple adversity types or to physical and sexual abuse [23, 24]. Our observation that cumulative exposure across childhood and adulthood relates to older ageing profiles extends this literature, aligning with recent evidence that life-course adversity is associated with elevated cardiovascular disease risk [27]. By evaluating childhood and adulthood adversity jointly and incorporating NMR-based metabolomic ageing indices, our study adds to this body of work.

Early life adversities may exert long-lasting effects on health, potentially through mechanisms such as epigenetic modifications, more so than later life adverse events [42]. In relation to adversity and mental health, the timing of childhood adverse events is also important [43, 44]. Windows of vulnerability potentially occur during periods of rapid development, and synaptic pruning during childhood and adolescence might unmask underlying predispositions. However, our findings suggest that for biological ageing markers, both early life as well as later life adversity are associated with worse outcomes. This finding aligns with prior research suggesting that cumulative trauma exposure exerts a greater impact on the development of psychopathology [45, 46]. Our study identified mostly older biological ageing profiles in individuals with greater breadth and severity of adverse events in childhood and in adulthood, apart from physical abuse, which was associated with greater grip strength in adulthood. We speculate that this association reflects a compensatory defensive mechanism in adults experiencing physical abuse.

Previously, we reported shorter telomeres in adults with a history of childhood adversity, primarily in clinical cohorts with severe mental disorders [47]. In contrast, here we analysed data from a large community-based sample. Compared to the general population, individuals with severe mental disorders are more likely to report adversities [48, 49]. Both in the current and our prior study, telomere length was measured using qPCR, a validated method for assessing average telomere length [50]. However, qPCR does not capture the number of critically short telomeres or cell-specific variation. Our findings suggest that the frailty index and MileAge delta may better capture the possible impact of adversity on biological ageing.

Although associations with molecular and functional ageing markers were modest in size, they were generally directionally consistent and increased with cumulative exposure. Given the high prevalence of adversity, small differences in biological ageing may translate into meaningful increases in morbidity and healthcare burden at the population level. Of note, because sociodemographic covariates may partially mediate associations between adversity and biological ageing, the fully adjusted model likely provides conservative estimates. However, differences between minimally adjusted and fully adjusted coefficients were modest, indicating that the observed associations were robust to covariate adjustment.

The two NMR-based ageing algorithms are trained on different targets: MileAge was trained on chronological age, whereas the metabolomic mortality profile was trained on mortality risk. They therefore may capture partially distinct biological processes: normative metabolomic ageing versus mortality-linked metabolic dysregulation. These differences have implications for construct validity. The metabolomic mortality profile score may be more sensitive to adversity-linked risk pathways, such as inflammation or metabolic dysregulation, whereas the MileAge clock may capture broader ageing processes. Future studies should evaluate these measures in independent cohorts and directly compare their performance across health outcomes.

Although mental health outcomes were not analysed in this study, our findings hold significant relevance for mental health research and practice, as they reinforce a robust body of evidence linking adversity exposure across the lifespan to poorer health trajectories. Exposure to adversity in both childhood and adulthood is associated with increased vulnerability to conditions such as depression and PTSD [51, 52]. Future research should investigate how molecular, clinical and functional markers of biological ageing—such as those assessed here—relate to mental health outcomes in individuals exposed to adversity and trauma. Such work may help elucidate how biological and psychological factors interact to shape health and well-being.

Certain limitations are worth noting. First, the cross-sectional study design precludes causal inference. Future studies could expand on our findings and investigate the links between adversity and changes in metabolomic ageing, frailty, telomere attrition and functional decline prospectively. Second, the current study was performed in a community-based population and not, for instance, exclusively in people with severe mental disorders who have a shorter life expectancy on average [53] and are more likely to experience higher levels of adversity [44, 49, 54]. Third, the UK Biobank is subject to a well-described healthy volunteer bias, which may attenuate associations and limit generalisability [55]. Fourth, whilst qPCR is often employed in epidemiological research, due to its ability to provide results using only a small amount of DNA [50], a better estimate of the relationship between telomeres and adversity could have been achieved using a measurement technique with a higher sensitivity to short telomeres. Fifth, the timing of adversity reports relative to biological ageing markers warrants consideration. Adverse events were reported retrospectively between 2016 and 2017, several years after baseline. Childhood adversity necessarily precedes biological ageing marker assessment, but adulthood adversity may have occurred either before or after baseline. Because the exact timing of adulthood events is unknown, some exposures could have taken place after biomarker assessment. As such, the possibility of reverse causality for adulthood adversity cannot be excluded. Sixth, the retrospective ascertainment of adversity exposure may introduce recall bias or underreporting. However, retrospective reports of childhood adversity have been shown, for instance, to predict adverse mental health outcomes at least as well as prospectively measured adversity [56]. Seventh, although some adversity domains share similar labels (e.g., emotional abuse), the childhood and adulthood items differ in wording and context; they do not represent repeated measures of the same construct across time. Finally, we used a short version of the CTQ, and thus, may have missed details about childhood adverse events not captured in this version.

## Conclusions

Both childhood and adulthood adverse events were negatively associated with molecular, clinical and functional markers of biological ageing. Biological ageing across diverse pathways might form part of the mechanism linking early and later life adversities to poorer long-term health outcomes and lifespan.

## Supplementary Information

Additional File 1: Supplementary Tables S1-S14. Table S1—UK Biobank data fields. Table S2—Sample characteristics stratified by childhood trauma. Table S3—Childhood trauma analytical sample sizes. Table S4—Associations between exposure to multiple adverse/traumatic events in childhood and ageing markers. Table S5—Associations between adverse/traumatic events in childhood and ageing markers (ordinal). Table S6—Associations between childhood trauma items and ageing markers. Table S7—Sample characteristics stratified by adulthood trauma. Table S8—Adulthood trauma analytical sample sizes. Table S9—Associations between exposure to multiple adverse/traumatic events in adulthood and ageing markers. Table S10—Associations between adverse/traumatic events in adulthood and ageing markers (ordinal). Table S11—Associations between adulthood trauma items and ageing markers. Table S12—Child and adulthood trauma analytical sample sizes. Table S13—Associations between adverse/traumatic events across childhood and/or adulthood and ageing markers. Table S14—Simplified overview of results across analyses.

## Data Availability

The data used are available to all bona fide researchers for health-related research that is in the public interest, subject to an application process and approval criteria. Study materials are publicly available online at http://www.ukbiobank.ac.uk.

## Abbreviations

CI: Confidence interval
CTQ: Childhood Trauma Questionnaire
IQR: Interquartile range
LASSO: Least Absolute Shrinkage and Selection Operator
MHQ: Mental Health Questionnaire
NMR: Nuclear magnetic resonance
PTSD: Post-traumatic stress disorder
qPCR: Quantitative polymerase chain reaction
SD: Standard deviation
T/S ratio: Telomere repeat copy number to single-copy gene ratio
