# Supplementary material for: Adverse events in both childhood and adulthood are associated with molecular, clinical and functional markers of ageing
Source: BMC Med. 2026 Apr 28;24:252. doi: 10.1186/s12916-026-04815-x (PMC13122998; doi:10.1186/s12916-026-04815-x)
Supplement: Supplementary file 1 — Additional File 1: Supplementary Tables S1-S14. Table S1—UK Biobank data fields. Table S2—Sample characteristics stratified by childhood trauma. Table S3—Childhood trauma analytical sample sizes. Table S4—Associations between exposure to multiple adverse/traumatic events in childhood and ageing markers. Table S5—Associations between adverse/traumatic events in childhood and ageing markers (ordinal). Table S6—Associations between childhood trauma items and ageing markers. Table S7—Sample characteristics stratified by adulthood trauma. Table S8—Adulthood trauma analytical sample sizes. Table S9—Associations between exposure to multiple adverse/traumatic events in adulthood and ageing markers. Table S10—Associations between adverse/traumatic events in adulthood and ageing markers (ordinal). Table S11—Associations between adulthood trauma items and ageing markers. Table S12—Child and adulthood trauma analytical sample sizes. Table S13—Associations between adverse/traumatic events across childhood and/or adulthood and ageing markers. Table S14—Simplified overview of results across analyses. [file 12916_2026_4815_MOESM1_ESM.docx]

**Additional file 1**

This material accompanies the article

**Adverse events in both childhood and adulthood are associated with molecular, clinical and functional markers of ageing**

Monica Aas^1*^, Thole H Hoppen^2^, Nexhmedin Morina^2^, Shiyu Zhang^3^, Bin Li^3^, Vid Mlakar^1^ and Julian Mutz^1*^

**Table of contents:**

[1. UK Biobank data fields 2](#_Toc214457428)

[2. Sample characteristics childhood trauma 4](#_Toc214457429)

[3. Analytical sample sizes childhood trauma 5](#_Toc214457430)

[4. Multiple adverse/traumatic events in childhood 6](#_Toc214457431)

[5. Adverse/traumatic events in childhood (ordinal) 7](#_Toc214457432)

[6. Item-specific adverse/traumatic events in childhood 8](#_Toc214457433)

[7. Sample characteristics adulthood trauma 9](#_Toc214457434)

[8. Analytical sample sizes adulthood trauma 10](#_Toc214457435)

[9. Multiple adverse/traumatic events in adulthood 11](#_Toc214457436)

[10. Adverse/traumatic events in adulthood (ordinal) 12](#_Toc214457437)

[11. Item-specific adverse/traumatic events in adulthood 13](#_Toc214457438)

[12. Analytical sample sizes child and adulthood trauma 14](#_Toc214457439)

[13. Adverse/traumatic events in child and/or adulthood 15](#_Toc214457440)

[14. Overview of results 16](#_Toc214457441)

# 1. UK Biobank data fields

| **Table S1.** UK Biobank data fields | |
| --- | --- |
| Data field ID | Variable name |
| Adverse events |  |
| 20487 | Felt hated by family member as a child |
| 20488 | Physically abused by family as a child |
| 20489 | Felt loved as a child |
| 20490 | Sexually molested as a child |
| 20491 | Someone to take to doctor when needed as a child |
| 20521 | Belittlement by partner or ex-partner as an adult |
| 20522 | Been in a confiding relationship as an adult |
| 20523 | Physical violence by partner or ex-partner as an adult |
| 20524 | Sexual interference by partner/ex-partner without consent (adult) |
| 20525 | Able to pay rent/mortgage as an adult |
| Covariates / misc. |  |
| 31 | Sex |
| 34 | Year of birth |
| 52 | Month of birth |
| 53 | Date of attending assessment centre |
| 54 | UK Biobank assessment centre |
| 738 | Average total household income before tax |
| 6138 | Qualifications |
| 20400 | Date of completing mental health questionnaire |
| 21000 | Ethnic background |
| 22189 | Townsend deprivation index at recruitment |
| Metabolomic ageing |  |
| 40000 | Date of death |
| [Category 220](https://biobank.ndph.ox.ac.uk/ukb/label.cgi?id=220) | NMR metabolomics |
| Frailty index |  |
| 134 | Number of self-reported cancers |
| 135 | Number of self-reported non-cancer illnesses |
| 136 | Number of operations, self-reported |
| 137 | Number of treatments/medications taken |
| 1200 | Sleeplessness / insomnia |
| 1930 | Miserableness |
| 1970 | Nervous feelings |
| 2020 | Loneliness, isolation |
| 2050 | Frequency of depressed mood in last 2 weeks |
| 2080 | Frequency of tiredness / lethargy in last 2 weeks |
| 2178 | Overall health rating |
| 2188 | Long-standing illness, disability or infirmity |
| 2227 | Other eye problems |
| 2247 | Hearing difficulty/problems |
| 2296 | Falls in the last year |
| 2316 | Wheeze or whistling in the chest in last year |
| 2335 | Chest pain or discomfort |
| 2443 | Diabetes diagnosed by doctor |
| 2453 | Cancer diagnosed by doctor |
| 2463 | Fractured/broken bones in last 5 years |
| 2473 | Other serious medical condition/disability diagnosed by doctor |
| 3786 | Age asthma diagnosed |
| 6148^*^ | Eye problems/disorders |
| 6149^*^ | Mouth/teeth dental problems |
| 6150^*^ | Vascular/heart problems diagnosed by doctor |
| 6152^*^ | Blood clot, DVT, bronchitis, emphysema, asthma, rhinitis, eczema, allergy diagnosed by doctor |
| 6153^*^ | Medication for cholesterol, blood pressure, diabetes, or take exogenous hormones |
| 6159^*^ | Pain type(s) experienced in last month |
| 6177^*^ | Medication for cholesterol, blood pressure or diabetes |
| 20002^*^ | Non-cancer illness code, self-reported |
| Telomere length |  |
| 22192 | Z-adjusted T/S log |
| Grip strength |  |
| 47 | Hand grip strength (right) |
| 46 | Hand grip strength (left) |
| 1707 | Handedness (chirality/laterality) |
| *Note:* ^*^ indicates array variables. | |

# 2. Sample characteristics childhood trauma

| **Table S2.** Sample characteristics stratified by childhood trauma | | | |
| --- | --- | --- | --- |
|  | Childhood trauma | |  |
|  | No  (*N*=90,491) | Yes  (*N*=63,066) | Full sample  (*N*=153,557) |
| MileAge delta, mean (SD)^1^ | -0.06 (3.74) | 0.01 (3.80) | -0.03 (3.76) |
| Mortality profile, mean (SD)^1^ | -45.11 (0.52) | -45.10 (0.52) | -45.11 (0.52) |
| Frailty index, mean (SD)^1^ | 0.10 (0.06) | 0.12 (0.07) | 0.11 (0.07) |
| T/S ratio, mean (SD)^1^ | 0.05 (0.99) | 0.06 (0.99) | 0.05 (0.99) |
| Grip strength, mean (SD)^1^ | 32.43 (10.95) | 32.48 (11.13) | 32.45 (11.03) |
| Age, mean (SD) | 56.86 (7.67) | 55.76 (7.77) | 56.41 (7.73) |
| Sex |  |  |  |
| Female | 50894 (56.2%) | 35578 (56.4%) | 86472 (56.3%) |
| Male | 39597 (43.8%) | 27488 (43.6%) | 67085 (43.7%) |
| Ethnicity |  |  |  |
| White | 88549 (97.9%) | 60189 (95.4%) | 148738 (96.9%) |
| Mixed | 292 (0.3%) | 500 (0.8%) | 792 (0.5%) |
| Black | 357 (0.4%) | 710 (1.1%) | 1067 (0.7%) |
| Asian | 570 (0.6%) | 691 (1.1%) | 1261 (0.8%) |
| Chinese | 118 (0.1%) | 231 (0.4%) | 349 (0.2%) |
| Other | 344 (0.4%) | 493 (0.8%) | 837 (0.5%) |
| Missing^2^ | 261 (0.3%) | 252 (0.4%) | 513 (0.3%) |
| Highest qualification |  |  |  |
| None | 5922 (6.5%) | 4522 (7.2%) | 10444 (6.8%) |
| O levels/GCSEs/CSEs | 21113 (23.3%) | 14782 (23.4%) | 35895 (23.4%) |
| A levels/NVQ/HND/HNC^3^ | 21047 (23.3%) | 15030 (23.8%) | 36077 (23.5%) |
| Degree | 41588 (46.0%) | 28120 (44.6%) | 69708 (45.4%) |
| Missing^2^ | 821 (0.9%) | 612 (1.0%) | 1433 (0.9%) |
| Household income^4^ |  |  |  |
| Very low | 10056 (11.1%) | 8769 (13.9%) | 18825 (12.3%) |
| Low | 18971 (21.0%) | 13290 (21.1%) | 32261 (21.0%) |
| Medium | 23519 (26.0%) | 16597 (26.3%) | 40116 (26.1%) |
| High | 21818 (24.1%) | 14380 (22.8%) | 36198 (23.6%) |
| Very high | 6804 (7.5%) | 4302 (6.8%) | 11106 (7.2%) |
| Missing^2^ | 9323 (10.3%) | 5728 (9.1%) | 15051 (9.8%) |
| Townsend deprivation |  |  |  |
| Q1 | 21842 (24.1%) | 12961 (20.6%) | 34803 (22.7%) |
| Q2 | 20451 (22.6%) | 12599 (20.0%) | 33050 (21.5%) |
| Q3 | 18965 (21.0%) | 12673 (20.1%) | 31638 (20.6%) |
| Q4 | 16964 (18.7%) | 13119 (20.8%) | 30083 (19.6%) |
| Q5 | 12162 (13.4%) | 11626 (18.4%) | 23788 (15.5%) |
| Missing^2^ | 107 (0.1%) | 88 (0.1%) | 195 (0.1%) |
| *Note:* Numbers shown are counts and percentages unless indicated otherwise. SD = standard deviation. GCSEs = general certificate of secondary education; CSE = certificate of secondary education; NVQ = national vocational qualification; HND = higher national diploma; HNC = higher national certificate. ^1^ Sample sizes for health and ageing markers: *n* = 69,451 (MileAge delta), 4260 (mortality profile), 153,387 (frailty index), 144,968 (T/S ratio) and 153,055 (grip strength). ^2^ Missing data may also include “do not know” or “prefer not to answer”. ^3^ Also includes 'other professional qualifications'. ^4^ Annual household income groups: very low (<£18,000), low (£18,000–£30,999), middle (£31,000–£51,999), high (£52,000–£100,000) and very high (>£100,000). | | | |

# 3. Analytical sample sizes childhood trauma

| **Table S3.** Childhood trauma analytical sample sizes | | | | | |
| --- | --- | --- | --- | --- | --- |
| Childhood trauma | MileAge delta | Mortality profile | Frailty index | Telomere length | Grip strength |
| No | 41138 (59.2%) | 2584 (60.7%) | 90412 (58.9%) | 85479 (59.0%) | 90201 (58.9%) |
| Yes | 28313 (40.8%) | 1676 (39.3%) | 62975 (41.1%) | 59489 (41.0%) | 62854 (41.1%) |
| 0 | 41138 (59.2%) | 2584 (60.7%) | 90412 (58.9%) | 85479 (59.0%) | 90201 (58.9%) |
| 1 | 15800 (22.7%) | 987 (23.2%) | 34869 (22.7%) | 32925 (22.7%) | 34813 (22.7%) |
| 2 | 7068 (10.2%) | 397 (9.3%) | 15853 (10.3%) | 14994 (10.3%) | 15817 (10.3%) |
| 3 | 3627 (5.2%) | 204 (4.8%) | 8161 (5.3%) | 7719 (5.3%) | 8148 (5.3%) |
| 4 | 1404 (2.0%) | 70 (1.6%) | 3181 (2.1%) | 2987 (2.1%) | 3169 (2.1%) |
| 5 | 414 (0.6%) | 18 (0.4%) | 911 (0.6%) | 864 (0.6%) | 907 (0.6%) |
| None | 41138 (59.2%) | 2584 (60.7%) | 90412 (58.9%) | 85479 (59.0%) | 90201 (58.9%) |
| One | 15800 (22.7%) | 987 (23.2%) | 34869 (22.7%) | 32925 (22.7%) | 34813 (22.7%) |
| Multiple | 12513 (18.0%) | 689 (16.2%) | 28106 (18.3%) | 26564 (18.3%) | 28041 (18.3%) |
| Sum score^1^ | 0.70 (1.04) | 0.65 (0.99) | 0.71 (1.05) | 0.71 (1.05) | 0.71 (1.05) |
| Weighted score^1^ | 1.24 (1.96) | 1.13 (1.83) | 1.25 (1.97) | 1.25 (1.97) | 1.25 (1.97) |
| Specific items | | | | | |
| Abuse, physical | 57400 (81.0%) | 3589 (82.5%) | 127000 (81.0%) | 119995 (81.0%) | 126735 (81.0%) |
| Yes | 13471 (19.0%) | 763 (17.5%) | 29730 (19.0%) | 28102 (19.0%) | 29663 (19.0%) |
| Abuse, emotional | 59969 (84.7%) | 3734 (85.8%) | 132162 (84.4%) | 124906 (84.4%) | 131875 (84.4%) |
| Yes | 10873 (15.3%) | 619 (14.2%) | 24475 (15.6%) | 23102 (15.6%) | 24428 (15.6%) |
| Abuse, sexual | 64195 (91.5%) | 3952 (91.8%) | 141628 (91.2%) | 133820 (91.2%) | 141329 (91.2%) |
| Yes | 5998 (8.5%) | 353 (8.2%) | 13613 (8.8%) | 12874 (8.8%) | 13580 (8.8%) |
| Neglect, emotional | 66581 (94.3%) | 4128 (95.2%) | 147045 (94.2%) | 138942 (94.2%) | 146731 (94.2%) |
| Yes | 4019 (5.7%) | 210 (4.8%) | 8971 (5.8%) | 8487 (5.8%) | 8952 (5.8%) |
| Neglect, physical | 55073 (77.8%) | 3439 (79.1%) | 121258 (77.5%) | 114623 (77.5%) | 120997 (77.5%) |
| Yes | 15689 (22.2%) | 906 (20.9%) | 35203 (22.5%) | 33236 (22.5%) | 35132 (22.5%) |
| *Note:* Numbers shown are counts and percentages unless indicated otherwise. ^1^ mean and standard deviation. | | | | | |

# 4. Multiple adverse/traumatic events in childhood

| **Table S4.** Associations between exposure to multiple adverse/traumatic events in childhood and ageing markers | | | | | | | | |
| --- | --- | --- | --- | --- | --- | --- | --- | --- |
|  | Model 1 (adj. age and sex) | | | | Model 2 (full adjustment) | | | |
|  | *β* | 95% CI | | *p* | *β* | 95% CI | | *p* |
| MileAge delta | | | | | | | | |
| None | Reference | | | | | | | |
| One | 0.009 | -0.010 | 0.027 | 0.359 | 0.009 | -0.010 | 0.027 | 0.359 |
| Multiple | 0.036 | 0.016 | 0.056 | 0.002 | 0.034 | 0.014 | 0.054 | 0.002 |
| Metabolomic profile | | | | | | | | |
| None | Reference | | | | | | | |
| One | 0.038 | -0.025 | 0.101 | 0.260 | 0.036 | -0.026 | 0.098 | 0.260 |
| Multiple | 0.077 | 0.005 | 0.149 | 0.144 | 0.041 | -0.030 | 0.113 | 0.260 |
| Frailty index | | | | | | | | |
| None | Reference | | | | | | | |
| One | 0.189 | 0.178 | 0.200 | <0.001 | 0.176 | 0.166 | 0.187 | <0.001 |
| Multiple | 0.461 | 0.450 | 0.473 | <0.001 | 0.422 | 0.411 | 0.434 | <0.001 |
| Telomere length | | | | | | | | |
| None | Reference | | | | | | | |
| One | -0.001 | -0.014 | 0.011 | 0.963 | 0.000 | -0.012 | 0.013 | 0.963 |
| Multiple | 0.027 | 0.014 | 0.041 | <0.001 | 0.031 | 0.018 | 0.045 | <0.001 |
| Grip strength | | | | | | | | |
| None | Reference | | | | | | | |
| One | 0.007 | -0.001 | 0.015 | 0.131 | -0.001 | -0.009 | 0.007 | 0.867 |
| Multiple | 0.034 | 0.025 | 0.042 | <0.001 | 0.014 | 0.005 | 0.022 | 0.004 |
| *Note:* CI = confidence interval. Model 1–adjusted for chronological age and sex; Model 2–adjusted for chronological age, sex, ethnicity, highest educational/professional qualification, annual gross household income and Townsend deprivation index. *P*-values shown are corrected for multiple testing using the Benjamini–Hochberg procedure (across exposure levels and models, separately for each ageing marker and childhood and adulthood exposures). Sample sizes reported in Table S2. | | | | | | | | |

# 5. Adverse/traumatic events in childhood (ordinal)

| **Table S5.** Associations between adverse/traumatic events in childhood and ageing markers (ordinal) | | | | | | | | |
| --- | --- | --- | --- | --- | --- | --- | --- | --- |
|  | Model 1 (adj. age and sex) | | | | Model 2 (full adjustment) | | | |
|  | *β* | 95% CI | | *p* | *β* | 95% CI | | *p* |
| MileAge delta | | | | | | | | |
| None | Reference | | | | | | | |
| 1 | 0.009 | -0.010 | 0.027 | 0.356 | 0.009 | -0.010 | 0.027 | 0.356 |
| 2 | 0.027 | 0.002 | 0.052 | 0.068 | 0.026 | 0.001 | 0.052 | 0.068 |
| 3 | 0.040 | 0.006 | 0.074 | 0.065 | 0.038 | 0.004 | 0.072 | 0.068 |
| 4 | 0.029 | -0.024 | 0.082 | 0.356 | 0.027 | -0.027 | 0.080 | 0.356 |
| 5 | 0.175 | 0.079 | 0.272 | 0.003 | 0.170 | 0.073 | 0.267 | 0.003 |
| Metabolomic profile | | | | | | | | |
| None | Reference | | | | | | | |
| 1 | 0.038 | -0.025 | 0.101 | 0.422 | 0.036 | -0.026 | 0.098 | 0.422 |
| 2 | 0.107 | 0.016 | 0.197 | 0.207 | 0.080 | -0.010 | 0.169 | 0.324 |
| 3 | 0.081 | -0.041 | 0.203 | 0.422 | 0.054 | -0.066 | 0.175 | 0.493 |
| 4 | -0.079 | -0.282 | 0.124 | 0.493 | -0.170 | -0.371 | 0.031 | 0.324 |
| 5 | -0.018 | -0.414 | 0.378 | 0.931 | -0.162 | -0.554 | 0.229 | 0.493 |
| Frailty index | | | | | | | | |
| None | Reference | | | | | | | |
| 1 | 0.189 | 0.178 | 0.200 | <0.001 | 0.177 | 0.166 | 0.187 | <0.001 |
| 2 | 0.354 | 0.340 | 0.369 | <0.001 | 0.327 | 0.313 | 0.342 | <0.001 |
| 3 | 0.517 | 0.498 | 0.537 | <0.001 | 0.475 | 0.455 | 0.494 | <0.001 |
| 4 | 0.693 | 0.662 | 0.724 | <0.001 | 0.632 | 0.602 | 0.663 | <0.001 |
| 5 | 1.028 | 0.971 | 1.084 | <0.001 | 0.925 | 0.869 | 0.981 | <0.001 |
| Telomere length | | | | | | | | |
| None | Reference | | | | | | | |
| 1 | -0.001 | -0.014 | 0.011 | 0.945 | 0.000 | -0.012 | 0.013 | 0.958 |
| 2 | 0.018 | 0.001 | 0.035 | 0.051 | 0.021 | 0.004 | 0.038 | 0.031 |
| 3 | 0.037 | 0.014 | 0.059 | 0.007 | 0.041 | 0.019 | 0.064 | 0.003 |
| 4 | 0.033 | -0.003 | 0.068 | 0.089 | 0.040 | 0.004 | 0.075 | 0.046 |
| 5 | 0.090 | 0.025 | 0.155 | 0.017 | 0.096 | 0.031 | 0.161 | 0.013 |
| Grip strength | | | | | | | | |
| None | Reference | | | | | | | |
| 1 | 0.007 | -0.001 | 0.015 | 0.123 | -0.001 | -0.009 | 0.007 | 0.873 |
| 2 | 0.018 | 0.007 | 0.029 | 0.003 | 0.003 | -0.008 | 0.013 | 0.715 |
| 3 | 0.048 | 0.034 | 0.063 | <0.001 | 0.027 | 0.012 | 0.041 | <0.001 |
| 4 | 0.059 | 0.036 | 0.082 | <0.001 | 0.027 | 0.005 | 0.050 | 0.031 |
| 5 | 0.095 | 0.053 | 0.138 | <0.001 | 0.047 | 0.005 | 0.090 | 0.040 |
| *Note:* CI = confidence interval. Model 1–adjusted for chronological age and sex; Model 2–adjusted for chronological age, sex, ethnicity, highest educational/professional qualification, annual gross household income and Townsend deprivation index. *P*-values shown are corrected for multiple testing using the Benjamini–Hochberg procedure (across exposure levels and models, separately for each ageing marker and childhood and adulthood exposures). Sample sizes reported in Table S2. | | | | | | | | |

# 6. Item-specific adverse/traumatic events in childhood

| **Table S6.** Associations between childhood trauma items and ageing markers | | | | | | | | |
| --- | --- | --- | --- | --- | --- | --- | --- | --- |
|  | Model 1 (adj. age and sex) | | | | Model 2 (full adjustment) | | | |
|  | *β* | 95% CI | | *p* | *β* | 95% CI | | *p* |
| MileAge delta | | | | | | | | |
| Abuse, physical | 0.033 | 0.014 | 0.052 | 0.002 | 0.033 | 0.014 | 0.052 | 0.002 |
| Abuse, emotional | 0.038 | 0.017 | 0.058 | 0.002 | 0.036 | 0.015 | 0.056 | 0.002 |
| Abuse, sexual | 0.030 | 0.004 | 0.057 | 0.047 | 0.030 | 0.003 | 0.056 | 0.047 |
| Neglect, emotional | 0.010 | -0.022 | 0.041 | 0.609 | 0.007 | -0.025 | 0.039 | 0.661 |
| Neglect, physical | 0.019 | 0.001 | 0.036 | 0.055 | 0.016 | -0.001 | 0.034 | 0.086 |
| Metabolomic profile | | | | | | | | |
| Abuse, physical | 0.043 | -0.024 | 0.110 | 0.642 | 0.025 | -0.041 | 0.091 | 0.655 |
| Abuse, emotional | -0.015 | -0.088 | 0.058 | 0.865 | -0.036 | -0.108 | 0.036 | 0.642 |
| Abuse, sexual | 0.109 | 0.015 | 0.202 | 0.224 | 0.066 | -0.026 | 0.159 | 0.642 |
| Neglect, emotional | 0.057 | -0.062 | 0.175 | 0.642 | 0.004 | -0.114 | 0.122 | 0.947 |
| Neglect, physical | 0.028 | -0.035 | 0.090 | 0.642 | 0.003 | -0.059 | 0.065 | 0.947 |
| Frailty index | | | | | | | | |
| Abuse, physical | 0.291 | 0.280 | 0.302 | <0.001 | 0.269 | 0.258 | 0.280 | <0.001 |
| Abuse, emotional | 0.412 | 0.400 | 0.424 | <0.001 | 0.380 | 0.368 | 0.392 | <0.001 |
| Abuse, sexual | 0.301 | 0.286 | 0.317 | <0.001 | 0.280 | 0.265 | 0.296 | <0.001 |
| Neglect, emotional | 0.357 | 0.338 | 0.376 | <0.001 | 0.283 | 0.264 | 0.301 | <0.001 |
| Neglect, physical | 0.341 | 0.330 | 0.351 | <0.001 | 0.306 | 0.295 | 0.316 | <0.001 |
| Telomere length | | | | | | | | |
| Abuse, physical | 0.033 | 0.020 | 0.046 | <0.001 | 0.037 | 0.024 | 0.050 | <0.001 |
| Abuse, emotional | 0.017 | 0.004 | 0.031 | 0.018 | 0.021 | 0.007 | 0.035 | 0.005 |
| Abuse, sexual | 0.022 | 0.004 | 0.040 | 0.018 | 0.027 | 0.010 | 0.045 | 0.005 |
| Neglect, emotional | 0.035 | 0.014 | 0.056 | 0.004 | 0.033 | 0.012 | 0.055 | 0.005 |
| Neglect, physical | 0.009 | -0.003 | 0.020 | 0.159 | 0.009 | -0.003 | 0.020 | 0.159 |
| Grip strength | | | | | | | | |
| Abuse, physical | -0.012 | -0.021 | -0.004 | 0.004 | -0.022 | -0.030 | -0.014 | <0.001 |
| Abuse, emotional | 0.049 | 0.040 | 0.058 | <0.001 | 0.034 | 0.025 | 0.043 | <0.001 |
| Abuse, sexual | 0.012 | 0.001 | 0.024 | 0.040 | 0.001 | -0.011 | 0.012 | 0.893 |
| Neglect, emotional | 0.089 | 0.075 | 0.103 | <0.001 | 0.052 | 0.038 | 0.066 | <0.001 |
| Neglect, physical | 0.036 | 0.029 | 0.044 | <0.001 | 0.022 | 0.014 | 0.029 | <0.001 |
| *Note:* CI = confidence interval. Model 1–adjusted for chronological age and sex; Model 2–adjusted for chronological age, sex, ethnicity, highest educational/professional qualification, annual gross household income and Townsend deprivation index. *P*-values shown are corrected for multiple testing using the Benjamini–Hochberg procedure (across trauma items and models, separately for each ageing marker and childhood and adulthood exposures). Sample sizes reported in Table S2. | | | | | | | | |

# 7. Sample characteristics adulthood trauma

| **Table S7.** Sample characteristics stratified by adulthood trauma | | | |
| --- | --- | --- | --- |
|  | Adulthood trauma | |  |
|  | No  (*N*=69,953) | Yes  (*N*=80,895) | Full sample  (*N*=150,848) |
| MileAge delta, mean (SD)^1^ | -0.08 (3.74) | 0.01 (3.78) | -0.03 (3.76) |
| Mortality profile, mean (SD)^1^ | -45.12 (0.51) | -45.09 (0.54) | -45.10 (0.52) |
| Frailty index, mean (SD)^1^ | 0.10 (0.06) | 0.12 (0.07) | 0.11 (0.07) |
| T/S ratio, mean (SD)^1^ | 0.03 (0.99) | 0.07 (0.99) | 0.05 (0.99) |
| Grip strength, mean (SD)^1^ | 33.62 (11.13) | 31.51 (10.85) | 32.49 (11.03) |
| Age, mean (SD) | 56.79 (7.59) | 56.01 (7.80) | 56.38 (7.72) |
| Sex |  |  |  |
| Female | 35764 (51.1%) | 49065 (60.7%) | 84829 (56.2%) |
| Male | 34189 (48.9%) | 31830 (39.3%) | 66019 (43.8%) |
| Ethnicity |  |  |  |
| White | 68646 (98.1%) | 77495 (95.8%) | 146141 (96.9%) |
| Mixed | 269 (0.4%) | 520 (0.6%) | 789 (0.5%) |
| Black | 196 (0.3%) | 857 (1.1%) | 1053 (0.7%) |
| Asian | 340 (0.5%) | 876 (1.1%) | 1216 (0.8%) |
| Chinese | 86 (0.1%) | 254 (0.3%) | 340 (0.2%) |
| Other | 221 (0.3%) | 589 (0.7%) | 810 (0.5%) |
| Missing^2^ | 195 (0.3%) | 304 (0.4%) | 499 (0.3%) |
| Highest qualification |  |  |  |
| None | 3930 (5.6%) | 6181 (7.6%) | 10111 (6.7%) |
| O levels/GCSEs/CSEs | 15358 (22.0%) | 19816 (24.5%) | 35174 (23.3%) |
| A levels/NVQ/HND/HNC^3^ | 16232 (23.2%) | 19169 (23.7%) | 35401 (23.5%) |
| Degree | 33871 (48.4%) | 34902 (43.1%) | 68773 (45.6%) |
| Missing^2^ | 562 (0.8%) | 827 (1.0%) | 1389 (0.9%) |
| Household income^4^ |  |  |  |
| Very low | 5725 (8.2%) | 12637 (15.6%) | 18362 (12.2%) |
| Low | 13514 (19.3%) | 18106 (22.4%) | 31620 (21.0%) |
| Medium | 18608 (26.6%) | 20953 (25.9%) | 39561 (26.2%) |
| High | 19239 (27.5%) | 16582 (20.5%) | 35821 (23.7%) |
| Very high | 6398 (9.1%) | 4603 (5.7%) | 11001 (7.3%) |
| Missing^2^ | 6469 (9.2%) | 8014 (9.9%) | 14483 (9.6%) |
| Townsend deprivation |  |  |  |
| Q1 | 17953 (25.7%) | 16185 (20.0%) | 34138 (22.6%) |
| Q2 | 16470 (23.5%) | 15978 (19.8%) | 32448 (21.5%) |
| Q3 | 14719 (21.0%) | 16357 (20.2%) | 31076 (20.6%) |
| Q4 | 12649 (18.1%) | 16923 (20.9%) | 29572 (19.6%) |
| Q5 | 8085 (11.6%) | 15337 (19.0%) | 23422 (15.5%) |
| Missing^2^ | 77 (0.1%) | 115 (0.1%) | 192 (0.1%) |
| *Note:* Numbers shown are counts and percentages unless indicated otherwise. SD = standard deviation. GCSEs = general certificate of secondary education; CSE = certificate of secondary education; NVQ = national vocational qualification; HND = higher national diploma; HNC = higher national certificate. ^1^ Missing data for health and ageing markers: *n* = 68,126 (MileAge delta), 4205 (mortality profile), 150,690 (frailty index), 142,381 (T/S ratio) and 150,362 (grip strength). ^2^ Missing data may also include “do not know” or “prefer not to answer”. ^3^ Also includes 'other professional qualifications'. ^4^ Annual household income groups: very low (<£18,000), low (£18,000–£30,999), middle (£31,000–£51,999), high (£52,000–£100,000) and very high (>£100,000). | | | |

# 8. Analytical sample sizes adulthood trauma

| **Table S8.** Adulthood trauma analytical sample sizes | | | | | |
| --- | --- | --- | --- | --- | --- |
| Adulthood trauma | MileAge delta | Mortality profile | Frailty index | Telomere length | Grip strength |
| No | 31760 (46.6%) | 2122 (50.5%) | 69905 (46.4%) | 66063 (46.4%) | 69730 (46.4%) |
| Yes | 36366 (53.4%) | 2083 (49.5%) | 80785 (53.6%) | 76318 (53.6%) | 80632 (53.6%) |
| 0 | 31760 (46.6%) | 2122 (50.5%) | 69905 (46.4%) | 66063 (46.4%) | 69730 (46.4%) |
| 1 | 20978 (30.8%) | 1262 (30.0%) | 46673 (31.0%) | 44108 (31.0%) | 46577 (31.0%) |
| 2 | 9476 (13.9%) | 508 (12.1%) | 20979 (13.9%) | 19774 (13.9%) | 20929 (13.9%) |
| 3 | 3747 (5.5%) | 201 (4.8%) | 8387 (5.6%) | 7967 (5.6%) | 8387 (5.6%) |
| 4 | 1635 (2.4%) | 89 (2.1%) | 3604 (2.4%) | 3387 (2.4%) | 3596 (2.4%) |
| 5 | 530 (0.8%) | 23 (0.5%) | 1142 (0.8%) | 1082 (0.8%) | 1143 (0.8%) |
| None | 31760 (46.6%) | 2122 (50.5%) | 69905 (46.4%) | 66063 (46.4%) | 69730 (46.4%) |
| One | 20978 (30.8%) | 1262 (30.0%) | 46673 (31.0%) | 44108 (31.0%) | 46577 (31.0%) |
| Multiple | 15388 (22.6%) | 821 (19.5%) | 34112 (22.6%) | 32210 (22.6%) | 34055 (22.6%) |
| Sum score^1^ | 0.89 (1.07) | 0.80 (1.03) | 0.89 (1.07) | 0.89 (1.07) | 0.89 (1.07) |
| Specific items | | | | | |
| Abuse, physical | 61742 (87.2%) | 3859 (88.7%) | 136568 (87.2%) | 129018 (87.2%) | 136267 (87.2%) |
| Yes | 9053 (12.8%) | 492 (11.3%) | 20031 (12.8%) | 18960 (12.8%) | 19996 (12.8%) |
| Abuse, emotional | 53923 (76.2%) | 3399 (78.1%) | 119066 (76.0%) | 112476 (76.0%) | 118795 (76.0%) |
| Yes | 16880 (23.8%) | 953 (21.9%) | 37541 (24.0%) | 35498 (24.0%) | 37474 (24.0%) |
| Abuse, sexual | 66740 (94.3%) | 4141 (95.2%) | 147535 (94.2%) | 139429 (94.2%) | 147215 (94.2%) |
| Yes | 4057 (5.7%) | 210 (4.8%) | 9039 (5.8%) | 8520 (5.8%) | 9027 (5.8%) |
| Neglect, emotional | 47003 (68.0%) | 2994 (70.3%) | 103941 (67.9%) | 98261 (68.0%) | 103692 (67.9%) |
| Yes | 22155 (32.0%) | 1263 (29.7%) | 49038 (32.1%) | 46283 (32.0%) | 48953 (32.1%) |
| Hardship, economic | 59735 (85.4%) | 3778 (87.7%) | 131984 (85.3%) | 124706 (85.3%) | 131687 (85.3%) |
| Yes | 10199 (14.6%) | 528 (12.3%) | 22670 (14.7%) | 21416 (14.7%) | 22634 (14.7%) |
| *Note:* Numbers shown are counts and percentages unless indicated otherwise. ^1^ mean and standard deviation. | | | | | |

# 9. Multiple adverse/traumatic events in adulthood

| **Table S9.** Associations between exposure to multiple adverse/traumatic events in adulthood and ageing markers | | | | | | | | |
| --- | --- | --- | --- | --- | --- | --- | --- | --- |
|  | Model 1 (adj. age and sex) | | | | Model 2 (full adjustment) | | | |
|  | *β* | 95% CI | | *p* | *β* | 95% CI | | *p* |
| MileAge delta | | | | | | | | |
| None | Reference | | | | | | | |
| One | 0.020 | 0.002 | 0.037 | 0.107 | 0.017 | -0.001 | 0.034 | 0.126 |
| Multiple | 0.009 | -0.010 | 0.029 | 0.461 | 0.003 | -0.017 | 0.023 | 0.760 |
| Metabolomic profile | | | | | | | | |
| None | Reference | | | | | | | |
| One | 0.084 | 0.025 | 0.144 | 0.011 | 0.053 | -0.007 | 0.112 | 0.110 |
| Multiple | 0.125 | 0.056 | 0.195 | 0.002 | 0.056 | -0.014 | 0.127 | 0.116 |
| Frailty index | | | | | | | | |
| None | Reference | | | | | | | |
| One | 0.176 | 0.166 | 0.186 | <0.001 | 0.139 | 0.128 | 0.149 | <0.001 |
| Multiple | 0.447 | 0.436 | 0.459 | <0.001 | 0.375 | 0.363 | 0.386 | <0.001 |
| Telomere length | | | | | | | | |
| None | Reference | | | | | | | |
| One | -0.009 | -0.020 | 0.003 | 0.182 | -0.009 | -0.021 | 0.002 | 0.182 |
| Multiple | 0.011 | -0.002 | 0.024 | 0.182 | 0.009 | -0.004 | 0.022 | 0.182 |
| Grip strength | | | | | | | | |
| None | Reference | | | | | | | |
| One | 0.053 | 0.045 | 0.060 | <0.001 | 0.035 | 0.027 | 0.042 | <0.001 |
| Multiple | 0.087 | 0.078 | 0.095 | <0.001 | 0.053 | 0.045 | 0.062 | <0.001 |
| *Note:* CI = confidence interval. Model 1–adjusted for chronological age and sex; Model 2–adjusted for chronological age, sex, ethnicity, highest educational/professional qualification, annual gross household income and Townsend deprivation index. *P*-values shown are corrected for multiple testing using the Benjamini–Hochberg procedure (across exposure levels and models, separately for each ageing marker and childhood and adulthood exposures). Sample sizes reported in Table S7. | | | | | | | | |

# 10. Adverse/traumatic events in adulthood (ordinal)

| **Table S10.** Associations between adverse/traumatic events in adulthood and ageing markers (ordinal) | | | | | | | | |
| --- | --- | --- | --- | --- | --- | --- | --- | --- |
|  | Model 1 (adj. age and sex) | | | | Model 2 (full adjustment) | | | |
|  | *β* | 95% CI | | *p* | *β* | 95% CI | | *p* |
| MileAge delta | | | | | | | | |
| None | Reference | | | | | | | |
| 1 | 0.020 | 0.002 | 0.037 | 0.209 | 0.017 | -0.001 | 0.034 | 0.209 |
| 2 | 0.016 | -0.007 | 0.039 | 0.352 | 0.011 | -0.012 | 0.034 | 0.485 |
| 3 | -0.011 | -0.045 | 0.023 | 0.587 | -0.019 | -0.053 | 0.016 | 0.478 |
| 4 | -0.006 | -0.056 | 0.044 | 0.804 | -0.016 | -0.066 | 0.034 | 0.587 |
| 5 | 0.084 | -0.002 | 0.170 | 0.209 | 0.067 | -0.019 | 0.154 | 0.315 |
| Metabolomic profile | | | | | | | | |
| None | Reference | | | | | | | |
| 1 | 0.085 | 0.025 | 0.144 | 0.027 | 0.053 | -0.007 | 0.112 | 0.159 |
| 2 | 0.131 | 0.048 | 0.213 | 0.020 | 0.081 | -0.002 | 0.164 | 0.139 |
| 3 | 0.057 | -0.067 | 0.181 | 0.521 | -0.017 | -0.140 | 0.107 | 0.791 |
| 4 | 0.205 | 0.023 | 0.387 | 0.091 | 0.064 | -0.118 | 0.246 | 0.566 |
| 5 | 0.299 | -0.053 | 0.650 | 0.159 | 0.117 | -0.231 | 0.466 | 0.566 |
| Frailty index | | | | | | | | |
| None | Reference | | | | | | | |
| 1 | 0.177 | 0.166 | 0.187 | <0.001 | 0.140 | 0.130 | 0.150 | <0.001 |
| 2 | 0.352 | 0.338 | 0.365 | <0.001 | 0.292 | 0.279 | 0.305 | <0.001 |
| 3 | 0.509 | 0.489 | 0.529 | <0.001 | 0.436 | 0.416 | 0.456 | <0.001 |
| 4 | 0.720 | 0.690 | 0.749 | <0.001 | 0.616 | 0.587 | 0.646 | <0.001 |
| 5 | 0.978 | 0.927 | 1.029 | <0.001 | 0.831 | 0.780 | 0.882 | <0.001 |
| Telomere length | | | | | | | | |
| None | Reference | | | | | | | |
| 1 | -0.009 | -0.020 | 0.003 | 0.242 | -0.009 | -0.021 | 0.003 | 0.242 |
| 2 | -0.001 | -0.016 | 0.015 | 0.928 | -0.002 | -0.017 | 0.014 | 0.928 |
| 3 | 0.015 | -0.007 | 0.038 | 0.268 | 0.014 | -0.009 | 0.037 | 0.278 |
| 4 | 0.033 | -0.001 | 0.067 | 0.187 | 0.029 | -0.005 | 0.063 | 0.236 |
| 5 | 0.134 | 0.075 | 0.193 | <0.001 | 0.130 | 0.072 | 0.189 | <0.001 |
| Grip strength | | | | | | | | |
| None | Reference | | | | | | | |
| 1 | 0.053 | 0.045 | 0.060 | <0.001 | 0.035 | 0.027 | 0.043 | <0.001 |
| 2 | 0.078 | 0.068 | 0.088 | <0.001 | 0.048 | 0.038 | 0.058 | <0.001 |
| 3 | 0.090 | 0.076 | 0.105 | <0.001 | 0.057 | 0.042 | 0.072 | <0.001 |
| 4 | 0.106 | 0.084 | 0.128 | <0.001 | 0.060 | 0.038 | 0.082 | <0.001 |
| 5 | 0.169 | 0.131 | 0.207 | <0.001 | 0.105 | 0.067 | 0.143 | <0.001 |
| *Note:* CI = confidence interval. Model 1–adjusted for chronological age and sex; Model 2–adjusted for chronological age, sex, ethnicity, highest educational/professional qualification, annual gross household income and Townsend deprivation index. *P*-values shown are corrected for multiple testing using the Benjamini–Hochberg procedure (across exposure levels and models, separately for each ageing marker and childhood and adulthood exposures). Sample sizes reported in Table S7. | | | | | | | | |

# 11. Item-specific adverse/traumatic events in adulthood

| **Table S11.** Associations between adulthood trauma items and ageing markers | | | | | | | | |
| --- | --- | --- | --- | --- | --- | --- | --- | --- |
|  | Model 1 (adj. age and sex) | | | | Model 2 (full adjustment) | | | |
|  | *β* | 95% CI | | *p* | *β* | 95% CI | | *p* |
| MileAge delta | | | | | | | | |
| Abuse, physical | -0.012 | -0.034 | 0.010 | 0.671 | -0.016 | -0.038 | 0.007 | 0.585 |
| Abuse, emotional | 0.002 | -0.015 | 0.020 | 0.996 | 0.000 | -0.018 | 0.018 | >0.999 |
| Abuse, sexual | -0.001 | -0.033 | 0.031 | >0.999 | -0.005 | -0.038 | 0.027 | 0.996 |
| Neglect, emotional | 0.013 | -0.003 | 0.029 | 0.585 | 0.008 | -0.008 | 0.024 | 0.671 |
| Economic hardship | 0.014 | -0.006 | 0.035 | 0.585 | 0.008 | -0.013 | 0.029 | 0.757 |
| Metabolomic profile | | | | | | | | |
| Abuse, physical | 0.004 | -0.077 | 0.084 | 0.925 | -0.048 | -0.128 | 0.032 | 0.425 |
| Abuse, emotional | 0.021 | -0.041 | 0.083 | 0.733 | 0.004 | -0.058 | 0.065 | 0.925 |
| Abuse, sexual | -0.008 | -0.128 | 0.112 | 0.925 | -0.069 | -0.188 | 0.050 | 0.425 |
| Neglect, emotional | 0.108 | 0.052 | 0.164 | <0.001 | 0.055 | -0.002 | 0.112 | 0.149 |
| Economic hardship | 0.194 | 0.117 | 0.271 | <0.001 | 0.117 | 0.039 | 0.195 | 0.011 |
| Frailty index | | | | | | | | |
| Abuse, physical | 0.308 | 0.295 | 0.321 | <0.001 | 0.265 | 0.252 | 0.278 | <0.001 |
| Abuse, emotional | 0.348 | 0.338 | 0.359 | <0.001 | 0.327 | 0.317 | 0.338 | <0.001 |
| Abuse, sexual | 0.387 | 0.367 | 0.406 | <0.001 | 0.344 | 0.325 | 0.363 | <0.001 |
| Neglect, emotional | 0.219 | 0.209 | 0.228 | <0.001 | 0.149 | 0.139 | 0.158 | <0.001 |
| Economic hardship | 0.290 | 0.278 | 0.303 | <0.001 | 0.209 | 0.196 | 0.222 | <0.001 |
| Telomere length | | | | | | | | |
| Abuse, physical | 0.022 | 0.007 | 0.037 | 0.019 | 0.020 | 0.005 | 0.035 | 0.025 |
| Abuse, emotional | 0.010 | -0.002 | 0.022 | 0.139 | 0.010 | -0.002 | 0.021 | 0.142 |
| Abuse, sexual | 0.019 | -0.002 | 0.041 | 0.129 | 0.022 | 0.001 | 0.044 | 0.088 |
| Neglect, emotional | -0.002 | -0.013 | 0.009 | 0.740 | -0.003 | -0.014 | 0.008 | 0.663 |
| Economic hardship | 0.022 | 0.008 | 0.037 | 0.018 | 0.017 | 0.002 | 0.031 | 0.059 |
| Grip strength | | | | | | | | |
| Abuse, physical | 0.026 | 0.016 | 0.035 | <0.001 | 0.010 | 0.000 | 0.019 | 0.048 |
| Abuse, emotional | 0.029 | 0.022 | 0.037 | <0.001 | 0.021 | 0.014 | 0.029 | <0.001 |
| Abuse, sexual | 0.035 | 0.021 | 0.049 | <0.001 | 0.017 | 0.003 | 0.031 | 0.023 |
| Neglect, emotional | 0.078 | 0.071 | 0.085 | <0.001 | 0.047 | 0.040 | 0.055 | <0.001 |
| Economic hardship | 0.085 | 0.075 | 0.094 | <0.001 | 0.051 | 0.042 | 0.060 | <0.001 |
| *Note:* CI = confidence interval. Model 1–adjusted for chronological age and sex; Model 2–adjusted for chronological age, sex, ethnicity, highest educational/professional qualification, annual gross household income and Townsend deprivation index. *P*-values shown are corrected for multiple testing using the Benjamini–Hochberg procedure (across trauma items and models, separately for each ageing marker and childhood and adulthood exposures). Sample sizes reported in Tables S7. | | | | | | | | |

# 12. Analytical sample sizes child and adulthood trauma

| **Table S12.** Child and adulthood trauma analytical sample sizes | | | | | |
| --- | --- | --- | --- | --- | --- |
| Cross-classification | MileAge delta | Mortality profile | Frailty index | Telomere length | Grip strength |
| Neither | 21921 (32.7%) | 1446 (35.1%) | 48284 (32.6%) | 45627 (32.6%) | 48156 (32.6%) |
| Childhood | 9464 (14.1%) | 638 (15.5%) | 20745 (14.0%) | 19618 (14.0%) | 20697 (14.0%) |
| Adulthood | 17691 (26.4%) | 1053 (25.5%) | 38843 (26.3%) | 36736 (26.3%) | 38762 (26.3%) |
| Both | 17889 (26.7%) | 987 (23.9%) | 40086 (27.1%) | 37846 (27.1%) | 40018 (27.1%) |
| *Note:* Numbers shown are counts and percentages. | | | | | |

# 13. Adverse/traumatic events in child and/or adulthood

| **Table S13.** Associations between adverse/traumatic events and ageing markers | | | | | | | | |
| --- | --- | --- | --- | --- | --- | --- | --- | --- |
|  | Model 1 (adj. age and sex) | | | | Model 2 (full adjustment) | | | |
|  | *β* | 95% CI | | *p* | *β* | 95% CI | | *p* |
| MileAge delta | | | | | | | | |
| Neither | Reference | | | | | | | |
| Childhood | 0.001 | -0.023 | 0.025 | 0.981 | 0.002 | -0.022 | 0.026 | 0.981 |
| Adulthood | 0.000 | -0.020 | 0.020 | 0.981 | -0.003 | -0.023 | 0.017 | 0.981 |
| Both | 0.032 | 0.012 | 0.051 | 0.010 | 0.028 | 0.008 | 0.048 | 0.021 |
| Metabolomic profile | | | | | | | | |
| Neither | Reference | | | | | | | |
| Childhood | 0.017 | -0.063 | 0.097 | 0.813 | 0.007 | -0.072 | 0.086 | 0.858 |
| Adulthood | 0.071 | 0.003 | 0.139 | 0.083 | 0.027 | -0.041 | 0.095 | 0.654 |
| Both | 0.135 | 0.065 | 0.204 | <0.001 | 0.083 | 0.013 | 0.153 | 0.059 |
| Frailty index | | | | | | | | |
| Neither | Reference | | | | | | | |
| Childhood | 0.213 | 0.199 | 0.227 | <0.001 | 0.206 | 0.192 | 0.220 | <0.001 |
| Adulthood | 0.199 | 0.187 | 0.211 | <0.001 | 0.154 | 0.143 | 0.166 | <0.001 |
| Both | 0.494 | 0.483 | 0.506 | <0.001 | 0.434 | 0.422 | 0.445 | <0.001 |
| Telomere length | | | | | | | | |
| Neither | Reference | | | | | | | |
| Childhood | 0.011 | -0.005 | 0.028 | 0.342 | 0.013 | -0.004 | 0.029 | 0.342 |
| Adulthood | -0.004 | -0.018 | 0.009 | 0.510 | -0.007 | -0.020 | 0.006 | 0.367 |
| Both | 0.008 | -0.005 | 0.021 | 0.342 | 0.010 | -0.004 | 0.023 | 0.342 |
| Grip strength | | | | | | | | |
| Neither | Reference | | | | | | | |
| Childhood | -0.001 | -0.011 | 0.010 | 0.899 | -0.005 | -0.016 | 0.005 | 0.399 |
| Adulthood | 0.061 | 0.053 | 0.070 | <0.001 | 0.041 | 0.032 | 0.049 | <0.001 |
| Both | 0.071 | 0.063 | 0.080 | <0.001 | 0.041 | 0.032 | 0.050 | <0.001 |
| *Note:* CI = confidence interval. Model 1–adjusted for chronological age and sex; Model 2–adjusted for chronological age, sex, ethnicity, highest educational/professional qualification, annual gross household income and Townsend deprivation index. *P*-values shown are corrected for multiple testing using the Benjamini–Hochberg procedure (across exposure levels and models, separately for each ageing marker). Sample sizes reported in Table S11. | | | | | | | | |

# 14. Overview of results

| **Table S14.** Simplified overview of results | | | | | |
| --- | --- | --- | --- | --- | --- |
|  | MileAge delta | Mortality profile | Frailty index | Telomere length | Grip strength |
| Childhood | | | | | |
| Binary | √ | ~ | √ | √ | ~ |
| Multiple^1^ | √ | ~ | √ | √ | √ |
| Sum scores | √ | × | √ | √ | √ |
| Sum scores (cat.) | ~ | ~ | √ | ~ | ~ |
| Item-level | ~ | ~ | √ | ~ | ~ |
| Adulthood | | | | | |
| Binary | ~ | ~ | √ | × | √ |
| Multiple^1^ | × | ~ | √ | × | √ |
| Sum scores | × | ~ | √ | √ | √ |
| Sum scores (cat.) | ~ | ~ | √ | ~ | √ |
| Item-level | × | ~ | √ | ~ | √ |
| Child and adulthood | | | | | |
| Cross-classification | ~ | ~ | √ | × | ~ |
| *Note:* ^1^ comparison of multiple vs no exposures only. √ indicates consistent evidence of association with biological ageing marker; × indicates consistent lack of evidence of association with biological ageing marker; ~ indicates partial evidence of association with biological ageing marker (e.g., nominal association) or mixed findings. | | | | | |
